# Attitude and Perceptions of Healthcare workers regarding ionizing radiation

**DOI:** 10.1192/j.eurpsy.2024.1709

**Published:** 2024-08-27

**Authors:** A. Haddar, I. Sellami, M. A. Ghrab, M. Hajjaji, K. Jmal Hammami, M. L. Masmoudi

**Affiliations:** ^1^Occupational medicine, Hedi Chaker Hospital; ^2^Sfax University, Sfax, Tunisia

## Abstract

**Introduction:**

In operating rooms, the routine use of radiological procedures is commonplace. However, this essential tool brings about significant concerns for healthcare workers due to the associated radiological risks. Understanding healthcare workers’ attitudes and perceptions about ionizing radiation is crucial for addressing these concerns.

**Objectives:**

This study aims to assess the perceptions and concerns of healthcare workers regarding radiation risks and their practices in the operating room.

**Methods:**

A cross-sectional study was conducted in February and March 2023 among the operating room staff of Habib Bourguiba University Hospital in Sfax, Tunisia. We used a self-administered questionnaire that included socio-professional data. Self-assessment of exposure risk and protection level against ionizing radiation was evaluated on a scale from 0 to 10, and attitudes were assessed using a 5-item Likert scale.

**Results:**

Our study population consisted of 92 healthcare workers, with 54.3% being male. When asking operating room workers about the availability, accessibility, and quality of lead aprons, the median scores were 3 (IQR [0;6.5]), 2 (IQR [0;5]), and 2 (IQR [0;5]), respectively. The median self-assessment score for exposure risk was 8 (IQR [5.5; 10]), while the median self-assessment score for protection against ionizing radiation was 1 (IQR [0;3]). Sixty percent of the population had limited knowledge of the harmful effects of ionizing radiation, with a median self-assessment knowledge score of 1.5 (IQR [0;3]). Sixty-two percent reported concerns regarding radiological risks. In terms of practices, 44.5% of the staff maintained a distance from the radiation source during intraoperative radiography, and 21.7% used the apron for protection. Dosimeters were not used by any of the participants. Concerns level was associated with self-assessment of exposure risk (p = 0.027).

**Conclusions:**

In conclusion, awareness of the risks generates anxiety and concern among staff; however, it alone is insufficient to alter our practices. This underscores the imperative for a proactive approach in implementing robust safety measures and comprehensive training programs.

**Disclosure of Interest:**

None Declared

